# Sequestosome-1/p62 Mediates TLR4-Induced Inflammatory Program in Dendritic Cells Under Normoxic and Hypoxic Conditions

**DOI:** 10.1007/s00018-025-05989-y

**Published:** 2025-12-01

**Authors:** Federica Coppola, Sara Monaci, Alessandro Falsini, Irene Filippi, Carlo Aldinucci, Fabio Carraro, Antonella Naldini

**Affiliations:** 1 Department of Molecular and Developmental Medicine, University of Siena, Via Aldo Moro 2, 53100 Siena, Italy; 2 Department of Medical Biotechnologies, University of Siena, Via Aldo Moro 2, 53100 Siena, Italy

**Keywords:** SQSTM1/p62, TLR4, XRK3F2, Dendritic cells

## Abstract

Sequestosome-1/p62, a multifunctional adaptor protein, plays a critical role in NFκB signaling. In response to Toll-like receptor 4 (TLR4) activation, p62 facilitates NFκB activation via its interaction with RIP1, a process dependent on the p62 ZZ-domain. Dendritic cells (DCs), key mediators of immune responses, express a diverse array of TLRs, and their activation triggers DC maturation and pro-inflammatory cytokine secretion, including IL-1β, through NFκB signaling. Given that DCs encounter varying oxygen tensions during their lifespan, their adaptability to hypoxia is crucial. We previously demonstrated that p62 contributes to DC survival under hypoxic stress, through its crosstalk with ERK1/2. Here, we investigated the role of p62 in lipopolysaccharide (LPS)-induced inflammatory responses in DCs exposed to both normoxic and hypoxic environments. Our findings revealed that LPS induces p62 overexpression and enhanced its interaction with RIP1, regardless of oxygen levels. Furthermore, pharmacological inhibition of p62 ZZ-domain, using XRK3F2, significantly attenuated p62/RIP1 interaction, resulting in diminished NFκB activation and IL-1β production. These results highlight p62 as a critical regulator of TLR4-mediated inflammatory program in DCs in either physiological or pathological microenvironments and thus suggesting novel therapeutic targets in inflammation.

**Supplementary Information:**

The online version contains supplementary material available at 10.1007/s00018-025-05989-y.

## Introduction

The sequestosome-1/p62, a multifunctional adaptor protein, integrates diverse cellular signals, including those involved in inflammation and selective autophagy. Beyond its role in cellular component clearance, p62 acts as a molecular scaffold, facilitating interactions between key signaling proteins, by direct interaction with several binding partners [1, 2]. Notably, p62 is implicated in NFκB signaling, a critical pathway driving inflammatory responses. Previous studies have shown that p62-deficient mice exhibit defects in sustained NFκB activation during T cell differentiation [3], highlighting its importance in immune regulation. Indeed, three different p62 domains have been shown to participate in NFκB signaling: the N-terminal Phox and Bpem1 (PB1) domain, the TRAF6 binding sequence (TBS) and the central zinc finger (ZZ-) domain. Specifically, the latter has been shown to directly bind to the Receptor-interacting protein (RIP) 1 [4], which is mainly known as a downstream molecule of the TNFα receptor (TNFR)-1. Of note, in addition to its role in TNFR-1 signaling, RIP1 is also engaged in TIR-domain-containing adapter-inducing interferonβ (TRIF)-dependent pro-inflammatory pathways and NFκB activation in response to Toll Like receptor (TLR) 3 and TLR4 stimulation [5–7]. In the context of TLR4, both TRIF-RIP1 and MyD88-dependent pathways are essential for robust NFκB activation and cytokine production [8].

Dendritic cells (DCs), pivotal orchestrators of innate and adaptive immunity, express a broad repertoire of TLRs. Activation of MD-2/TLR4 by bacterial lipopolysaccharide (LPS) triggers DC maturation, which is characterized by enhanced antigen presentation and pro-inflammatory cytokine secretion, including IL-1β [9]. In this regard, the NFκB and NLRP3-inflammasome play a pivotal role. Indeed, NFκB not only promotes the expression of chemokines, cytokines, and cytokine precursors, such as pro-IL-1β, but it is also critical for upregulating NLRP3 expression [10]. In turn, the NLRP3-inflammasome mediates the maturation of pro-IL-1β into its biologically active form [11]. In addition to the NLRP3/IL-1β signaling, NFκB exerts a bidirectional crosstalk with the Hypoxia inducible factor (HIF)-1: it was reported that HIF-1α promotes NFκB activation while NFκB regulates HIF-1α transcription and stabilization. In this regard, previous studies have demonstrated HIF-1α involvement in innate immune responses and in DC physiology [12–14]. Given that DCs encounter varying oxygen (O_2_) tension during their lifespan, navigating from normoxic peripheral tissues [15], to hypoxic lymphoid organs and pathological microenvironments, their ability to adapt to hypoxia is essential [16–18]. However, the influence of hypoxia and HIF-1 on DC maturation and inflammatory properties remains controversial [19–21].

We have previously shown that the autophagic process, which is associated with p62 modulation, plays a critical role in DC adaptation to hypoxic stress, especially upon LPS-induced maturation [22], in terms of both cell survival and pro-inflammatory cytokine production [23]. Building on this, the present study aimed to investigate whether p62, through its interaction with RIP1, can directly contribute to NFκB activation and IL-1β secretion in DCs, thereby influencing their pro-inflammatory function, either in aerobic and hypoxic microenvironments. Here, we show that LPS enhances p62 expression and its interaction with RIP1 in DCs. Moreover, we here show that pharmacological inhibition of the p62 ZZ-domain, using XRK3F2 [24], disrupts p62/RIP1 interaction, attenuating NFκB activation and IL-1β production in mature DCs under both under normoxic and hypoxic conditions. These findings reveal a novel mechanism by which p62, via RIP1 engagement, regulates TLR4-mediated NFκB activation and IL-1β secretion in DCs, offering potential therapeutic targets for modulating inflammatory responses.

## Results

### LPS stimulation induces p62 and RIP1 upregulation and enhances their binding in human DCs

**Fig. 1:**
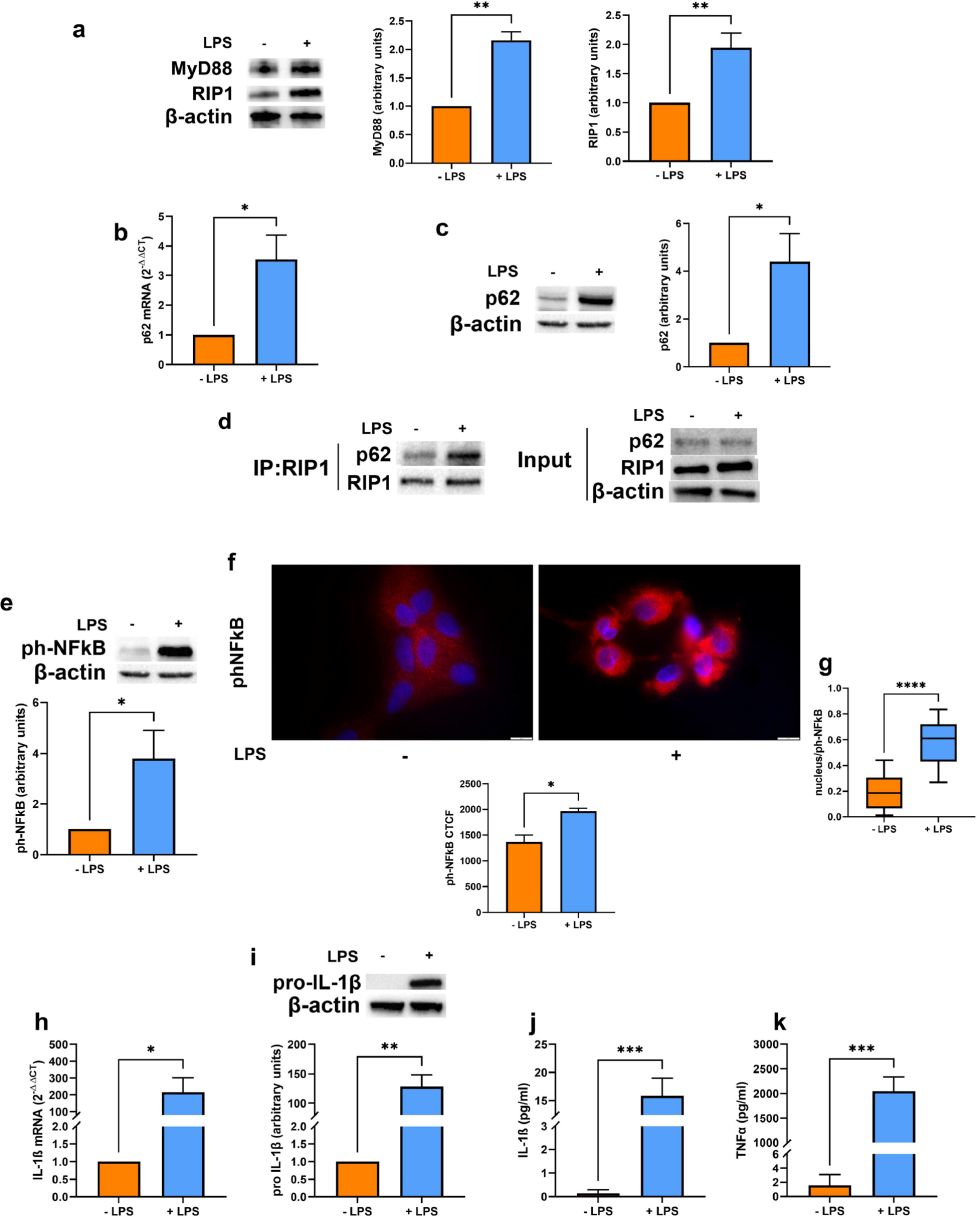
(**a**) MyD88 and RIP1 protein levels as determined by Western blotting, in DCs stimulated or not with LPS and exposed to normoxia (20% O_2_) for 24 h. (**b**) p62 mRNA and (**c**) protein levels, as determined by RT-qPCR and Western blotting respectively, in DCs stimulated or not with LPS and exposed to normoxia for 24 h. (**d**) Representative blot of co-immunoprecipitation of RIP1 with p62 and the relative input, in DCs stimulated or not with LPS and exposed to normoxia for 24 h. (**e** and **f**) phNFκB p65 protein levels, as shown by Western Blotting (**e**) and immunofluorescence assay (Scale bar: 10 μm), expressed as CTCF (**f**), in DCs stimulated or not with LPS and exposed to normoxia for 24 h. (**g**) phNFκB p65 nuclear localization, as detected by immunofluorescence assay (Scale bar: 10 μm) and expressed as Manders’ coefficient, in DCs stimulated or not with LPS and exposed to normoxia for 24 h. (**h**) IL-1β mRNA and (**i**) pro-IL-1β protein levels, as determined by RT-qPCR and Western blotting respectively, in DCs stimulated or not with LPS and exposed to normoxia for 24 h. (**j**) IL-1β and (**k**) TNFα secretion, as measured by ELISA assay, in DCs stimulated or not with LPS and exposed to normoxia for 24 h. All blots shown are representative of at least three independent experiments and β-actin was used as loading control. β-actin was used as a housekeeping gene for RT-qPCR analysis. * indicates statistically significant differences (*p* < 0.05)

We previously demonstrated that autophagy, which is associated with the modulation of p62 protein levels, plays a key role in DC physiology, under both normoxic and hypoxic conditions, especially upon LPS-induced maturation [23]. Thus, in the present paper, we decided to investigate whether p62 expression could be affected by LPS stimulation and how it could be involved in mature DC pro-inflammatory responses. First, we exposed DCs to a pO_2_ of 140 mmHg (normoxia) for 24 h, either in the presence or not of LPS, and examined the expression of the TLR4-downstream adaptors MyD88 and RIP1. As expected, LPS-induced maturation of DCs was associated with the upregulation of MyD88. In addition, LPS treatment resulted in a significant increase of RIP1 protein levels (Fig. 1a). Notably, LPS treatment also significantly increased p62 expression, at both the mRNA and protein levels (Fig. 1b and c), and enhanced its interaction with RIP1 (Fig. 1d). This interaction is known to be mediated by the p62 ZZ domain [4]. Furthermore, LPS-induced upregulation of p62 and its interaction with RIP1 correlated with increased NFκB p65 phosphorylation and nuclear translocation (Fig. 1e-g), consistent with enhanced pro-inflammatory signaling. Indeed, as expected, LPS stimulation also led to increased IL-1β mRNA (Fig. 1h) and pro-IL-1β intracellular levels (Fig. 1i), as well as to the enhancement of IL-1β and TNFα secretion (Fig. 1j and k). These data indicate that LPS treatment induces p62 overexpression along with the promotion of pro-inflammatory responses in DCs.

### p62 ZZ-domain inhibition disrupts p62/RIP1 interaction and impairs NFκB activation in LPS-stimulated DCs

**Fig. 2:**
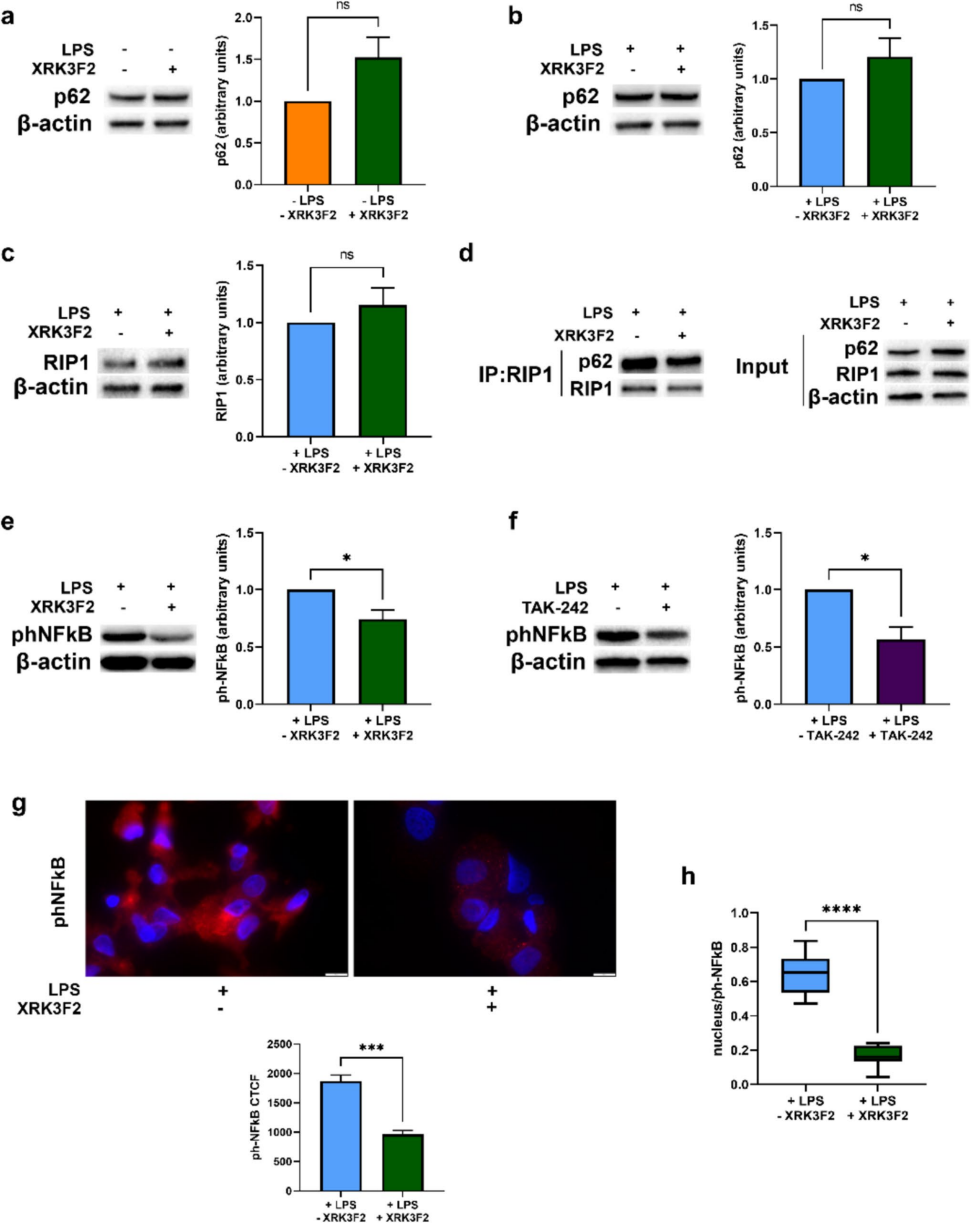
(**a**) p62 protein levels, as determined by Western blotting, in DCs exposed to normoxia for 24 h and treated or untreated in the last 6 h with XRK3F2. (**b**) p62 and (**c**) RIP1 protein levels, as determined by Western blotting, in LPS-stimulated DCs exposed to normoxia for 24 h and treated or untreated in the last 6 h with XRK3F2. (**d**) Representative blot of co-immunoprecipitation of RIP1 with p62 and the relative input, in LPS-stimulated DCs exposed to normoxia for 24 h and treated or untreated in the last 6 h with XRK3F2. (**e**) phNFκB p65 protein levels, as shown by Western Blotting, in LPS-stimulated DCs exposed to normoxia for 24 h and treated or untreated in the last 6 h with XRK3F2. (**f**) phNFκB p65 protein levels, as assessed by Western Blotting, after 24 h of exposure to normoxia of DCs pretreated or not for 1 h with TAK-242 and then stimulated with LPS. (**g**) phNFκB p65 protein levels and (**h**) phNFκB p65 nuclear localization, as determined by immunofluorescence assay (Scale bar: 10 μm), and expressed as CTCF and Manders’ coefficient respectively, in LPS-stimulated DCs exposed to normoxia for 24 h and treated or untreated in the last 6 h with XRK3F2. All blots shown are representative of at least three independent experiments and β-actin was used as loading control. * indicates statistically significant differences (*p* < 0.05)

To determine the molecular mechanism underlying p62 involvement in DC pro-inflammatory signals, we employed XRK3F2, a specific inhibitor of the p62 ZZ domain [27]. DCs, stimulated or not with LPS, were exposed to normoxia for 24 h and treated (where indicated) with XRK3F2 in the last 6 h, to minimize potential effects on autophagy-mediated degradation or de novo synthesis of p62. As shown in Fig. 2a and b, XRK3F2 treatment did not significantly affect p62 protein levels either in immature or in mature DCs. A similar result was observed for RIP1 expression in mature DCs (Fig. 2c). However, XRK3F2 strongly reduced p62/RIP1 interaction in LPS-stimulated DCs (Fig. 2d). This disruption of p62/RIP1 binding was accompanied by a significant decrease of NFκB p65 phosphorylation (Fig. 2e and g), comparable to the effect observed with the TLR4 inhibitor TAK242 (Fig. 2f). Moreover, XRK3F2 treatment reduced nuclear localization of phospho-p65 (NFκB) in mature DCs (Fig. 2h), suggesting that p62/RIP1 interaction is crucial for proper NFκB activation upon LPS-induced TLR4 activation.

### XRK3F2 treatment of LPS-stimulated DCs affects cytokine production and release

**Fig. 3:**
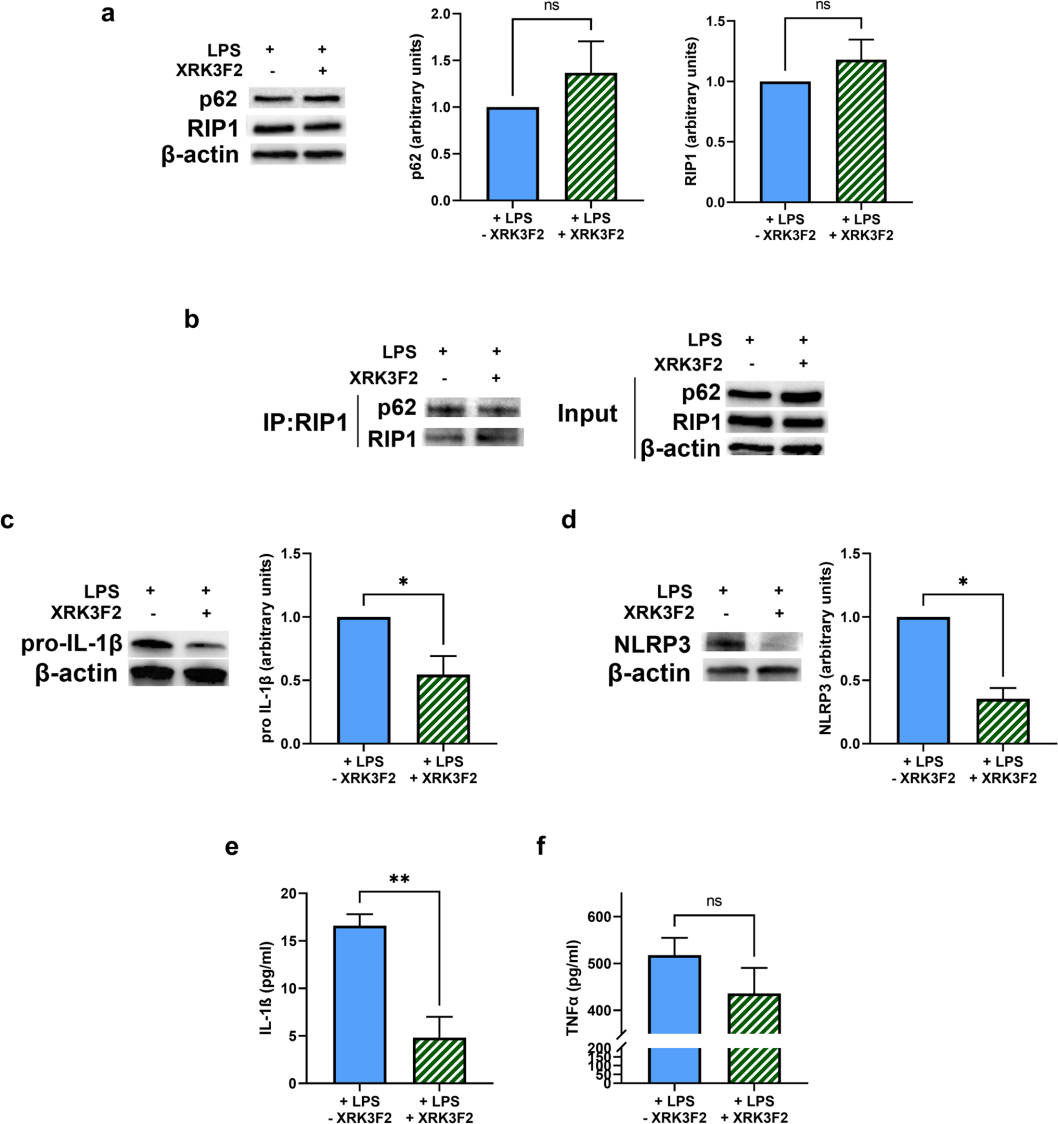
(**a**) p62 and RIP1 protein levels, as determined by Western blotting, in DCs exposed to normoxia for 24 h and treated or untreated throughout the incubation period with XRK3F2. (**b**) Representative blot of co-immunoprecipitation of RIP1 with p62 and the relative input, in LPS-stimulated DCs exposed to normoxia for 24 h and treated or untreated throughout the incubation period with XRK3F2. (**c**) pro-IL-1β and (**d**) NLRP3 protein levels, as shown by Western Blotting, in LPS-stimulated DCs exposed to normoxia for 24 h and treated or untreated throughout the incubation period with XRK3F2. (**e**) IL-1β and (**f**) TNFα secretion, as measured by ELISA assay, in LPS-stimulated DCs exposed to normoxia for 24 h and treated or untreated throughout the incubation period with XRK3F2. All blots shown are representative of at least three independent experiments and β-actin was used as loading control. * indicates statistically significant differences (*p* < 0.05)

To further assess the impact of p62 in pro-inflammatory functions, DCs were simultaneously treated with LPS and XRK3F2 for 24 h, to better appreciate the impact of this compound either on earlier events, such as NLRP3/inflammasome priming, and on events occurring throughout the entire incubation, including cytokine release. While XRK3F2 treatment did not significantly alter p62 or RIP1 expression (Fig. 3a), it reduced their interaction (Fig. 3b) albeit to a lesser extent than the 6-h treatment. More interestingly, XRK3F2-treated DCs exhibited lower intracellular pro-IL-1β levels (Fig. 3c), and reduced NLRP3 expression (Fig. 3D), leading to decreased secretion of mature IL-1β (Fig. 3e). A similar trend, though not statistically significant, was observed for TNFα secretion (Fig. 3f). The overall results support the hypothesis that that the p62/RIP1 interaction contributes to pro-inflammatory signaling in LPS-stimulated DCs.

### p62-targeting siRNA and autophagy inhibition by SAR405 result in IL-1β upregulation

**Fig. 4:**
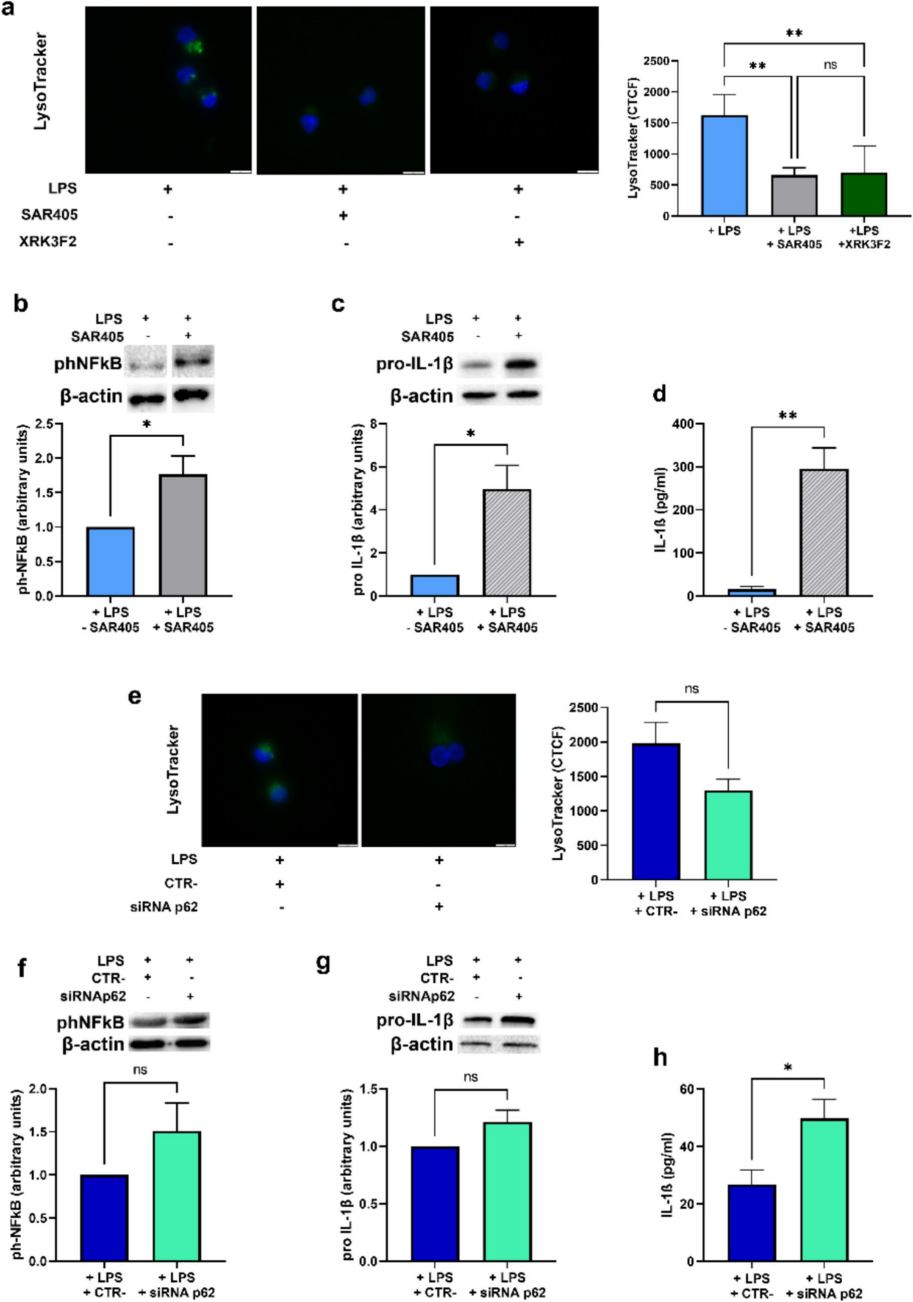
(**a**) Acidification of the autolysosomal compartment, determined by LysoTracker staining (Scale bar: 10 μm) and (**b**) phNFκB p65 ( protein levels, as determined by Western blotting, in LPS-stimulated DCs exposed to normoxia and treated, or untreated, during the last 6 h of incubation, either with SAR405 or XRK3F2. (**c**) pro-IL-1β protein levels and (**d**) IL-1β secretion, as measured by Western blotting and ELISA assay respectively, in DCs exposed to normoxia for 24 h and treated or untreated throughout the incubation period with SAR405. (**e**) Acidification of the autolysosomal compartment, determined by LysoTracker staining (Scale bar: 10 μm), (**f**) phNFκB p65 protein levels and (**g**) pro-IL-1β, as shown by Western Blotting, and (**h**) IL-1β secretion as measured by ELISA assay in LPS-stimulated DCs transfected either with CTR- or siRNAp62 and exposed to normoxia for 24 h. All blots shown are representative of at least three independent experiments and β-actin was used as loading control. * indicates statistically significant differences (*p* < 0.05)

We next decided to verify whether the effects observed upon XRK3F2 treatment were independent of its potential role in autophagy inhibition. To this end, LPS-stimulated DCs were treated, during the last 6 h of incubation, either with XRK3F2 or with the autophagy inhibitor SAR405 [23]. As shown in Fig. 4a, XRK3F2 treatment resulted in a significant reduction in the acidification of the autolysosomal compartment, indicating autophagy inhibition, comparable to that induced by SAR405. However, unlike XRK3F2 treatment (see Fig. 2e), SAR405 markedly increased NFκB p65 phosphorylation (Fig. 4b). Additionally, co-treatment with LPS and SAR405, throughout the entire incubation (24 h), was associated with a remarkable increase in pro-IL-1β protein levels and mature IL-1β secretion (Fig. 4c and d). Furthermore, DCs were transfected either with a control sequence or with a specific p62-targeting siRNA and stimulated with LPS for 24 h. As shown in Fig. 4e, p62 knockdown was associated to diminished autolysosomal acidification, even though not in a statistically significant manner. Moreover, p62 knockdown via siRNA did not produce statistically significant changes in NFκB p65 phosphorylation (Fig. 4f) or in pro-IL-1β protein levels (Fig. 4g). However, a significant increase in the release of mature IL-1β was observed in cells transfected with the p62-targeting siRNA compared to the controls (Fig. 4h). The latter results may be justified by the fact that inhibition of autophagy lead to a derangement of inflammatory dampening [23]. The overall results suggest that the inhibitory effects of XRK3F2 might be independent of autophagy inhibition and of other p62 domains, but that are specifically related to the ZZ one, and thus to p62/RIP1 interaction.

### p62/RIP1 interaction is involved in NFκB activation under hypoxia

**Fig. 5:**
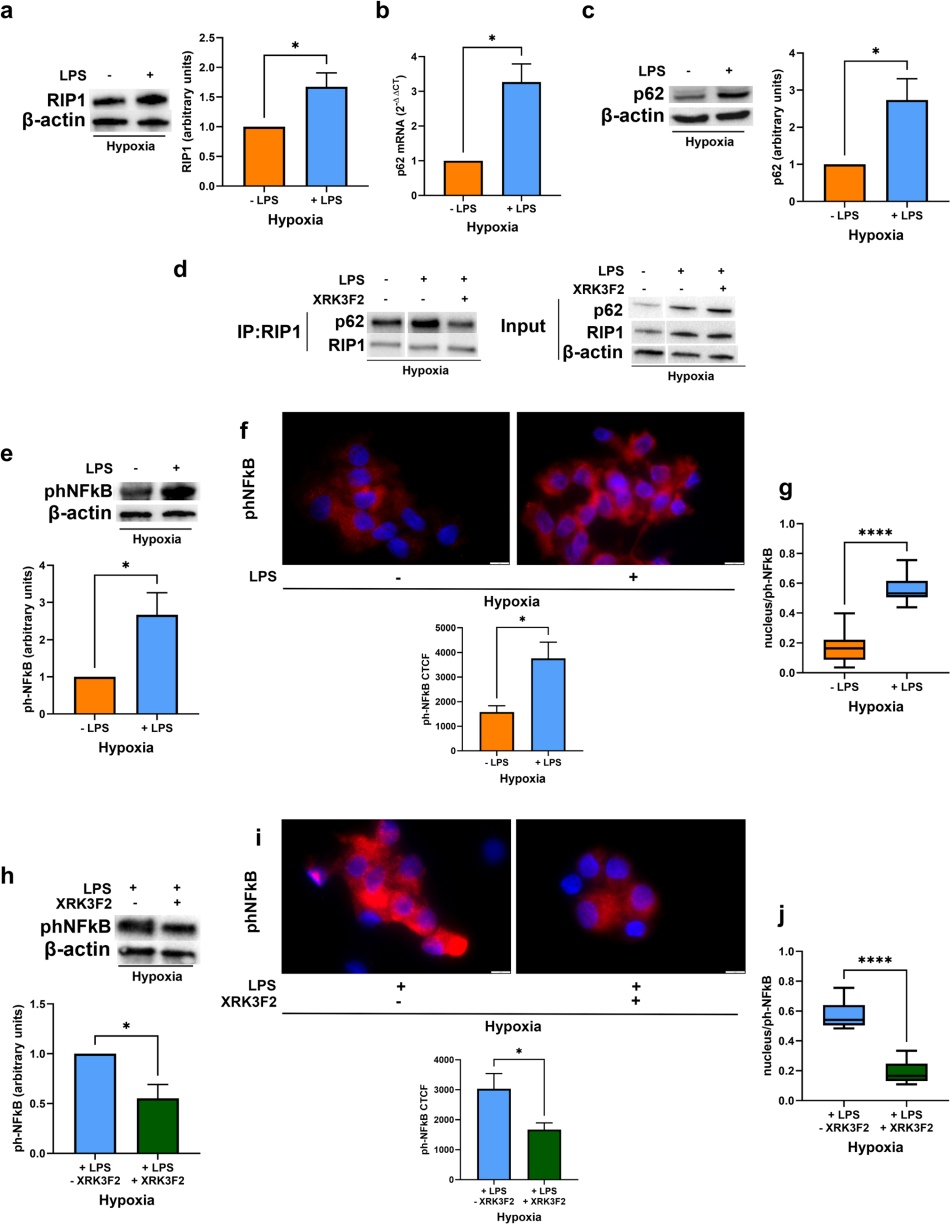
(**a**) RIP1 protein levels as determined by Western blotting, in DCs stimulated or not with LPS and exposed to hypoxia (2% O_2_) for 24 h. (**b**) p62 mRNA and (**c**) protein levels, as determined by RT-qPCR and Western blotting respectively, in DCs stimulated or not with LPS and exposed to hypoxia for 24 h. (**d**) Representative blot of co-immunoprecipitation of RIP1 with p62 and the relative input, in DCs, exposed to hypoxia for 24 h, in the presence or not of LPS and treated or untreated in the last 6 h with XRK3F2. (e and f) phNFκB p65 protein levels, as shown by Western Blotting (**e**) and immunofluorescence assay (Scale bar: 10 μm), expressed as CTCF (**f**), in DCs stimulated or not with LPS and exposed to hypoxia for 24 h. (**g**) phNFκB p65 nuclear localization, as detected by immunofluorescence assay (Scale bar: 10 μm) and expressed as Manders’ coefficient, in DCs stimulated or not with LPS and exposed to hypoxia for 24 h. (**h** and **i**) phNFκB p65 protein levels, as shown by Western Blotting (**h**) and immunofluorescence assay (Scale bar: 10 μm), expressed as CTCF (**i**), in LPS-stimulated DCs exposed to hypoxia for 24 h and treated or untreated in the last 6 h with XRK3F2. (**j**) phNFκB p65 nuclear localization, as detected by immunofluorescence assay (Scale bar: 10 μm) and expressed as Manders’ coefficient, in LPS-stimulated DCs exposed to hypoxia for 24 h and treated or untreated in the last 6 h with XRK3F2. All blots shown are representative of at least three independent experiments and β-actin was used as loading control. β-actin was used as a housekeeping gene for RT-qPCR analysis. * indicates statistically significant differences (*p* < 0.05)

Given that DCs migrate to hypoxic environments in vivo [19, 28, 29], we investigated the role of p62 in hypoxic DCs. DCs were exposed to hypoxia (pO_2_ of 14 mmHg ~2% O_2_) for 24 h in the presence or not of LPS and, where indicated, XRK3F2 was added in the last 6 h of incubation. Similarly to the aerobic conditions, LPS stimulation increased RIP1 protein levels (Fig. 5a), enhanced the expression of p62, as assessed by qRT-PCR and Western blotting (Fig. 5b and c) and promoted their interaction. Such effect was reverted by XRK3F2 treatment, as shown in Fig. 5d. Indeed, XRK3F2 disrupted p62/RIP1 interaction in mature DCs exposed to hypoxia. LPS also increased NFκB p65 phosphorylation and its nuclear translocation in hypoxic DCs (Fig. 5e-g), effects which were attenuated by XRK3F2 treatment (Fig. 5h-j). These data indicate that p62 overexpression and its interaction with RIP1 are critical for NFκB activation in both normoxic and hypoxic environments.

### XRK3F2 treatment impairs p62/RIP1 interaction affecting pro-inflammatory signals in hypoxic LPS-stimulated DCs

**Fig. 6:**
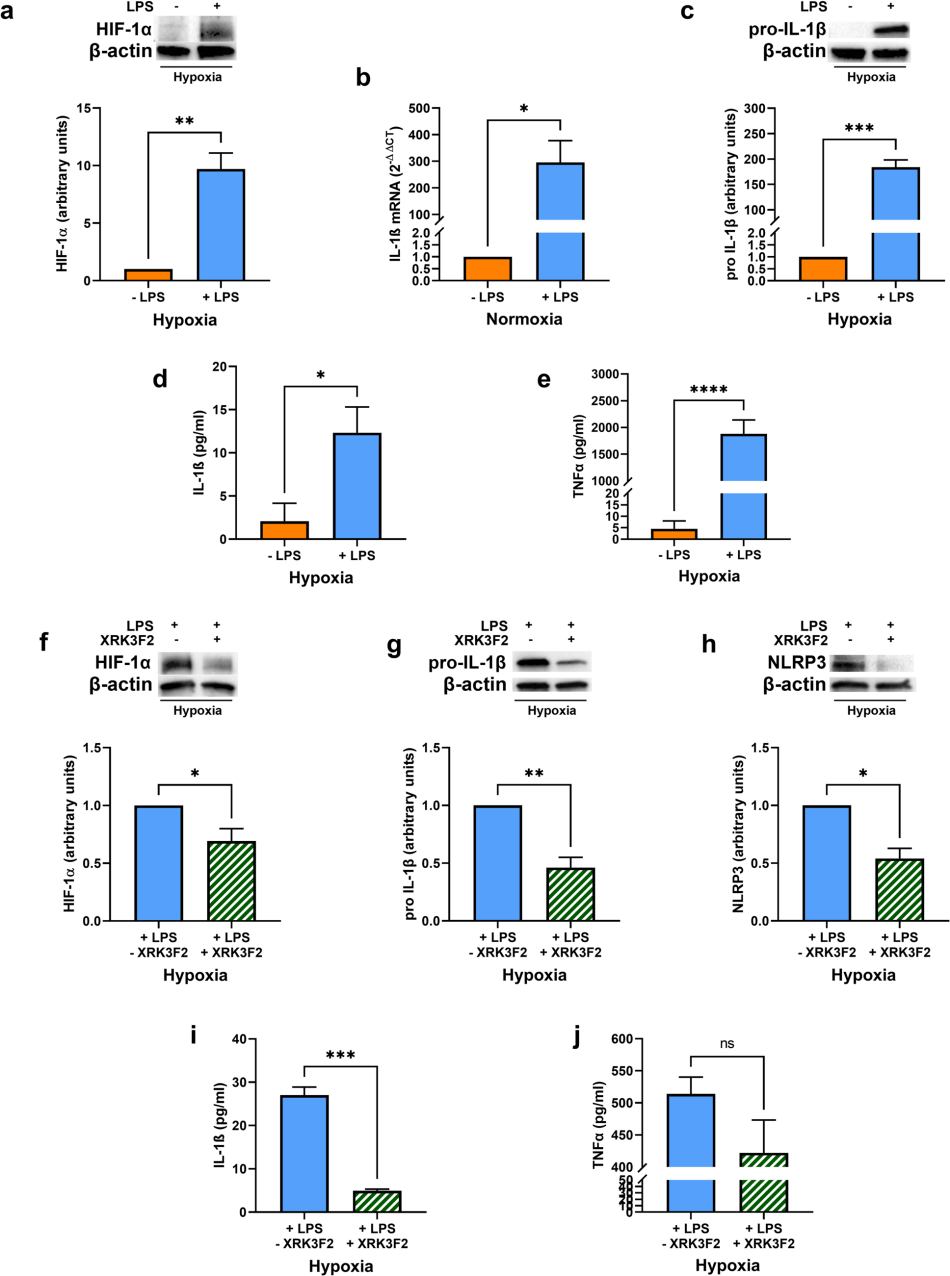
(**a**) HIF-1α protein levels as determined by Western blotting, in DCs stimulated or not with LPS and exposed to hypoxia for 24 h. (**b**) IL-1β mRNA and (**c**) pro-IL-1β protein levels, as determined by RT-qPCR and Western blotting respectively, in DCs stimulated or not with LPS and exposed to hypoxia for 24 h. (**d**) IL-1β and (**e**) TNFα secretion, as measured by ELISA assay, in DCs stimulated or not with LPS and exposed to hypoxia for 24 h. (**f**) HIF-1α, (**g**) pro-IL-1β and (**h**) NLRP3 protein levels, as shown by Western Blotting, in LPS-stimulated DCs exposed to hypoxia for 24 h and treated or untreated throughout the incubation period with XRK3F2. (**i**) IL-1β and (**j**) TNFα secretion, as measured by ELISA assay, in LPS-stimulated DCs exposed to hypoxia for 24 h and treated or untreated throughout the incubation period with XRK3F2. All blots shown are representative of at least three independent experiments and β-actin was used as loading control. β-actin was used as a housekeeping gene for RT-qPCR analysis. * indicates statistically significant differences (*p* < 0.05)

Thereafter, we evaluated the impact of p62 on the hypoxic signature and pro-inflammatory responses of mature DCs, by exposing DCs, either stimulated or not with LPS, to hypoxia for 24 h. LPS stimulation sustained hypoxia-induced HIF-1α accumulation (Fig. 6a), and, in parallel, increased IL-1β expression, at both RNA and protein level (Fig. 6b and c), as well as its secretion (Fig. 6d). A similar increase was observed for the release of TNFα (Fig. 6e). Notably, a 24 h treatment with XRK3F2 reverted such LPS-mediated effects, reducing HIF-1α and pro-IL-1β intracellular levels (Fig. 6f and g), as well as NLRP3 expression (Fig. 6h), which was paralleled by a significant reduction of mature IL-1β secretion (Fig. 6i). Finally, XRK3F2 treatment was associated also to diminished TNFα secretion, even though not in a statistically significant manner and in a lower extent when compared to IL-1β (Fig. 6j). These data suggest that the p62/RIP1 interaction influences pro-inflammatory responses and the hypoxic signature of LPS-stimulated DCs, further supporting its role in DC function under both normoxic and hypoxic conditions.

## Methods

### Human monocyte-derived DCs preparation, culture conditions and treatment

Human monocyte-derived DCs were generated from anonymous buffy obtained from the South-East Tuscany Blood Establishment (AOUS, Siena, Italy), as previously described [25]. This study was approved by Ethical Committee of Azienda Ospedaliera Universitaria Senese and University of Siena (CAVSE 17022020) and informed consent was obtained from all participants. Briefly, monocytes were isolated by Fycoll and Percoll gradient centrifugations of highly monocyte enriched blood (> 95% CD14 +). Then, monocytes were differentiated into DCs (> 90% CD1a + and < 5% CD14 +) by culturing for 6 days in RPMI 1640, supplemented with 10% FBS, penicillin (100 U/ml)/streptomycin (100 µg/ml) and 2 mM L-Glutamine (all purchased from Euroclone, Devon, UK) and with 50 ng/mL GM-CSF and 20 ng/mL IL-4 (purchased from PeproTech, Cranbury, NJ, USA). Endotoxin levels in all reagents were < 0.125 units/ml, as determined by the Limulus amebocyte lysate assay (Cambrex, East Rutherford, NJ, USA).

DCs were incubated for 24 h at 37 °C under either normoxia (21% O_2_, 5% CO_2_ and 74% N2), corresponding to a pO_2_ of ~ 140 mmHg, or hypoxia (2% O_2_, 5% CO_2_ and 94% N2), corresponding to a pO_2_ ~ 14 mmHg, by using a Coy O_2_ Controlled InVitro Glove-Box—Hypoxia Chamber (Coy Laboratory Products, Grass Lake, MI, USA). DC maturation was induced by adding 100 ng/mL of LPS (Sigma-Aldrich, St. Louis, MO, USA) to the culture medium for 24 h, as previously described [25]. Where indicated, DCs were treated with 5 µM XR3KF2 (Selleckchem, Houston, TX, USA) or 10 µM SAR405 (CliniSciences, Nanterre, France) for either the last 6 h or the entire 24-h incubation period. In some experiments, DCs were pretreated with 100 nM TAK242 (Sigma-Aldrich), a TLR4 inhibitor, for 2 h before LPS stimulation.

### RNA interference

p62 silencing was performed as previously described [31]. Briefly, a specific p62-targerting siRNA (NM_003900), siRNA p62 (SASI_Hs01_00118616) and a MISSION® siRNA Universal Negative Control #1 (SIC001) siRNA (CTR-), purchased from Sigma-Aldrich, St. Louis, MO, USA, were used at concentration of 46 nM. Transfection was performed by using Lipofectamine RNAi MAX (Invitrogen, Paisley, UK) and Lypofectamine-siRNA complexes were diluted in OPTI-MEM® (1X) (Gibco, Thermo Fisher Scientific, Cleveland, OH, USA). siRNA-Lipofectamine complexes, were added to the cells and DCs were incubated for 24 h under normoxia. Following transfection, the growth medium was replaced and DCs were exposed to normoxic conditions for 24 h.

### Western blot

DCs, treated or not as previously described, were lysed in RIPA buffer (Cell Signaling Technolo-gies, Danvers, MA, USA) containing protease inhibitors (Sigma-Aldrich, St. Louis, MO, USA). Equal amounts of total proteins for each sample were separated by 4–20% Mini-protean TGX gels (BioRad, Hercules, CA, USA) and transferred onto a nitrocellulose membrane (BioRad, Hercules, CA, USA). Membranes were blocked and incubated overnight, at 4 °C, with the following primary antibodies: MyD88 (Cell Signaling Technologies,Danvers, MA, 1:1000, Cat.n° 4283); RIP1 (Cell Signaling Technologies,Danvers, MA, 1:1000, Cat.n° 3493); SQSTM1/p62 (Cell Signaling Technologies,Danvers, MA, 1:1000, Cat.n° 5114); ph-p65 (NFκB) (Cell Signaling Technologies,Danvers, MA, 1:1000, Cat.n° 3033); pro-IL-1β (Cell Signaling Technologies,Danvers, MA, 1:1000, Cat.n° 12703); NLRP3 (Cell Signaling Technologies,Danvers, MA, 1:1000, Cat.n° 15101); HIF-1α (BD Biosciences, San Jose, CA, USA, 1:200 Cat.n◦ 610958); and b-actin (Sigma-Aldrich, St. Louis, MO, USA, 1:50000, Cat.n° A3854). After washing, membranes were incubated with horseradish peroxidase (HRP)-conjugated secondary anti-mouse I anti-rabbit antibodies (Cell Signaling Technologies, Danvers, MA, USA). Images were acquired by ChemiDoc™ MP System and densitometry analysis was performed with Image Lab software (BioRad, Hercules, CA, USA).

### RNA extraction and RT-qPCR

Total RNA was extracted using EuroGOLD™Trifast reagent (Euroclone, Devon, UK). cDNA was synthesized with iScript™cDNA Synthesis Kit (BioRad, Hercules, CA, USA) according to the manufacturer’s instructions. RT-qPCR was performed using SsoAdvanced™ Universal SYBR® Green Supermix (BioRad, Hercules, CA, USA). Relative mRNA expression levels of p62, were determined with CFX Duet Real Time-PCR System (BioRad, Hercules, CA, USA) and normalized to β-actin and calculated using the 2^−∆∆CT^ method.

### Immunoprecipitation assay

Immunoprecipitation assay was performed by using the Abcam kit (ab206996), accordingly to the manufacturer's instructions. Briefly, cells were lysed in 150 µL of RIPA buffer (Cell Signaling Technolo-gies, Danvers, MA, USA) supplemented with protease inhibitors (Sigma-Aldrich, St. Louis, MO, USA). Then Protein lysates (150 µg) were incubated overnight, at 4 °C with either the anti-RIP1 (Cell Signaling Technologies,Danvers, MA, 1:100, Cat.n° 3493) or the anti-normal IgG antibody (Cell Signaling Technologies,Danvers, MA, 1:1000, Cat.n° 7074). This step was followed by a 2 h incubation, at 4 °C, with protein A agarose beads. After washing, bound proteins were eluted and used for western blot analysis.

### Immunofluorescence analysis

DCs were seeded on 8-well coverglass slide (Sarstedt, Germany), at a concentration of 50.000 cells for each condition, fixed with methanol at -20 °C. and incubated overnight, at 4 °C (in a humidified chamber), with phospho-NFκB p65 (Ser536) primary antibody (Cell Signaling Technologies,Danvers, MA, 1:500, Cat.n° 3033). After washing, the secondary conjugated-antibody (Anti-rabbit DyLightTM 550 from Thermo Fisher Scientific, Cleveland, OH, USA, 1:250, Cat.n° 84541), was added and incubated for 1 h, at room temperature. Nuclei were stained with Hoechst 33342 (1 mg/ml, 1:1000, Fluka, Sigma Aldrich, Saint Louis, MO, USA). Images were acquired using an Olympus IX81 microscope (Olympus, Tokyo, Japan) and analyzed with imageJ software. Fluorescence intensity was expressed as corrected total cell fluorescence (CTCF) = integrated density—(area of selected cell X mean fluorescence of background readings). Co-localization analysis of phospho-NFκB and nuclei was performed with JACoP plugin, calculating Manders’ coefficient [26].

### Enzyme-linked immunosorbent assay (ELISA)

IL-1β and TNFα levels in cell culture supernatants were measured using ELISA kits (Cat. Nos. DY201 and DY210, respectively; R&D Systems, Minneapolis, MN, USA) according to the manufacturer's instructions. Briefly, 96-wells microplates were coated with capture antibodies, and standards and samples (properly diluted) were added to the plates and incubated at 37 ◦C for 2 h. After incubation and washing, biotinylated detection antibodies were added and plates were incubated at 37 ◦C for 1 h. After washing, streptavidin conjugated to horseradish peroxidase was added to each well. The reaction was developed by using TMB substrate, then stopped with sulphuric acid solution. Absorbance was measured at 450 nm with MULTISKAN (ThermoFisher Scientific, San Jose, CA, USA). To subtract high background signals, a reference measurement at 650 nm was performed.

### LysoTracker staining

LysoTracker staining was performed as previously described [22]. Briefly, cells were seeded onto 8-well coverglass slides (Sarstedt, Germany) and stimulated or unstimulated with LPS under normoxic conditions. Either XRK3F or SAR405, were added, where indicated, 6 h prior to the experiment's completion. After 24 h, autolysosomal acidification was detected by staining cells with the Lyso-ID Green Detection Kit (Enzo Life Sciences, Plymouth Meeting, PA, USA). Nuclear staining was performed with Hoechst 33342 (1 mg/ml, 1:1000, Fluka, Sigma Aldrich, Saint Louis, MO, USA). Images were acquired using an Olympus IX81 microscope (Olympus, Tokyo, Japan), with 60X magnification, and analyzed with imageJ software. Fluorescence intensity was expressed as CTCF.

### Statistical analysis

Data are shown as the mean ± standard error of the mean SEM of at least 3 independent experiments. Unpaired two-tailed student’s t-test was performed with Graph-Pad Prism (San Diego, CA, USA). p ≤ 0.05 was considered statistically significant.

## Discussion

In the present paper we propose a novel role for the adaptor protein p62 in regulating TLR4-mediated inflammatory responses in human monocyte-derived dendritic cells (DCs). We here showed, for the first time, that maturation of human DCs, induced by the MD-2/TLR4 ligand LPS, is associated with p62 overexpression and with the enhancement of its interaction with RIP1, a key signaling molecule downstream of TLR4. This interaction, mediated by the p62 ZZ-domain, contributes to NFκB activation and subsequent pro-inflammatory cytokine production, including IL-1β, in both normoxic and hypoxic environments.

We and others have previously shown that autophagy, in which p62 is strictly involved, plays a pivotal role in DCs, as it participates in their immune functions as well as in the modulation of cell viability [23, 30]. More recently, we have further investigated p62 pro-survival functions in hypoxic DCs, highlighting the importance of this protein in DC biology, since its ability to interact with other pathways beyond autophagy, including the ERK one [31]. Indeed, p62 is involved in the transduction of several intracellular signals, including those driving NFκB activation. It has been reported that p62 can promote NFκB activation and nuclear translocation, via direct interaction with TRAF6, MEKK3 and RIP1 [32, 33]. The importance of RIP1 in MyD88-independent TLR4 signaling, particularly in TRIF-dependent pathway leading to late-phase NFκB activation, is well established [8] and the dispensable role of the TIR domain–containing adaptor molecule MyD88 in TLR4 signaling transduction has been widely studied [34]. Accordingly, our data demonstrate that LPS stimulation not only increases MyD88 levels in human monocyte-derived DCs, but also upregulates RIP1, highlighting the cooperative effort of MyD88 and TRIF-dependent signals, which is required for proper pro-inflammatory cytokine secretion [35]. However, the effects of LPS treatment on immune cells are not restricted to the expression of canonical TLR4-downstream adaptors. As previously reported, activation of MD-2/TLR4 by LPS induces both p62 mRNA and protein level upregulation in macrophages [10]. In agreement with this work, we observed that DC maturation, induced by LPS, led to the enhancement of p62 expression at both mRNA and protein level. While the precise mechanism driving p62 upregulation in DCs remains to be fully elucidated, previous studies have identified NFκB and Nrf2 as transcriptional regulators involved in p62 overexpression [10, 36] and an a positive feedback loop, where NFκB promotes p62 expression, which in turn sustains NFκB activation, has been hypothesized in other cell types [37]. However, our study focuses on p62 ability to interact with RIP1, which depends on p62 ZZ-domain [4]. Interestingly, we reported that p62 upregulation was accompanied by the enhancement of its interaction with RIP1. As reported in previous works, p62/RIP1 interaction, induced by TNFR or IL-1R activation, leads to NFκB activation [4, 33]. Accordingly, we here observed that p62/RIP1 interaction promoted by TLR4 stimulation was associated with increased NFκB phosphorylation and nuclear translocation as well as with pro-IL-1β overexpression and enhanced secretion of fully mature IL-1β and TNFα. These data unveil a potential role for p62 in pro-inflammatory signals of mature DCs, since its MD-2/TLR4-induced overexpression and interaction with RIP1 are associated with NFκB activation and cytokine production.

The small molecule XRK3F2, which specifically targets p62 ZZ-domain [38, 39], was shown to impair TNFα-induced NFκB phosphorylation in bone marrow stromal cells from multiple myeloma patients [39]. Likewise, we found that XRK3F2 treatment of mature DCs affected NFκB phosphorylation. Of note, such decrease of NFκB phosphorylation was analogous to the TLR4 inhibitor (TAK242)-mediated one, further suggesting that p62, and in particular p62 ZZ-domain, may play a crucial role in NFκB signaling in LPS-stimulated DCs. Moreover, we observed that XRK3F2-dependent impairment of NFκB activation was not limited to its phosphorylation, but it also led to the reduction in its nuclear translocation. Such effects of XRK3F2 are not attributable to alterations in the expression levels neither of p62, as we and others have demonstrated [40], nor of RIP1. Reasonably, our findings hint that such effects are linked to a diminished interaction between these two proteins, which resulted upon ZZ-domain blockage by XRK3F2. In addition, we found out that decreased NFκB activation was accompanied by impaired pro-IL-1β expression in mature DCs. More interestingly, XRK3F2 also affected the secretion of fully mature IL-1β. Thus, we hypnotized that it may be related to defects in processing of the inactive pro-IL-1β. In these regards we observed that, along with NFκB phosphporylation, NLRP3 expression was downregulated upon XRK3F2 treatment. In agreement with our findings, several works have previously assessed the relevance of NFκB in NLRP3 overexpression and in keeping NLRP3-inflammasome activation [10, 41, 42]. However, concerning p62-NLRP3 crosstalk, a previous study has hypothesized that p62 overexpression may dampen inflammation by negatively regulating NLRP3-inflammasome activation via autophagic clearance of damaged mitochondria [10]. Nevertheless, it is crucial to note that, upon TLR4 activation, TRIF-RIP1-FADD-CASP8-dependent signaling mediates NLRP3-inflammasome alternative priming, as recently described in human monocytes [43]. Thus, we hypothesized that XRK3F2-mediated disruption of p62/RIP1 binding may be responsible for impaired alternative NLRP3-inflammasome priming in human monocyte-derived DCs. In support of this theory, we found out that treatment of mature DCs with XRK3F2 reduced TNFα secretion, albeit not significantly, and notably to a lower extent when compared to IL-1β. Of interest, these effects appear to be independent of autophagy inhibition, which is known to exacerbate inflammation [44]. Indeed, as indicated in a previous study, inhibition of autophagy through SAR405, a Class III PI3k/Vps34 targeting compound, induced the overexpression of pro-inflammatory cytokines [23]. Accordingly, in the present study, SAR405 treatment, unlike the inhibition of the p62 ZZ-domain, resulted in an increase in the pro-inflammatory properties of LPS-stimulated DCs (via upregulation of NFκB phosphorylation and IL-1β production), although XRK3F2 and SAR405 inhibited autophagy to the same extent. Furthermore, in our study we observed that the p62-targeting siRNA enhances the release of IL-1β. This may correlate with a partial, even though not significant, reduction of autophagy. In addition, our data are in agreement with a previous study, conducted in different experimental models, showing that full-length p62 knockout enhances inflammatory responses via NFκB activation [45]. These findings corroborate our hypothesis that p62 may be engaged in mature DC pro-inflammatory signals. Moreover, differences between p62 knockdown and ZZ-domain inhibition suggest that p62 potential involvement in NFκB activation and cytokine production, upon LPS-stimulation, is ZZ-domain-dependent. p62/RIP1 interaction may represent a key event in pro-inflammatory signal transduction downstream of TLR4 and in particular in MyD88-independent and TRIF-dependent signaling [8]. Indeed, we observed that TLR4 inhibition by TAK-242 was associated with a decrease of p62/RIP1 interaction (Fig. S1a) and that, in contrast to TAK-242, which reduced MyD88 protein levels (Fig. S1b), treatment with XRK3F2 did not affect MyD88 expression (Fig. S1c).

Notably, our findings demonstrate that the p62 contribution to MD-2/TLR4-dependent inflammatory program is crucial in both normoxic and hypoxic conditions, highlighting its relevance in several physiological and pathological settings. Indeed, DCs encounter varying O_2_ tensions during their lifespan, migrating from normoxic tissues to hypoxic lymphoid organs and inflammatory sites and it has been widely demonstrated that decreased O_2_ availability and HIF-1α markedly modulate DC survival and functions, with regards to their maturation stage [19, 20, 46, 47]. However, we reported XRK2F3 effects on hypoxic DCs were comparable to those observed under normoxia. Thus, we speculate that hypoxia may not affect this pathway, which appears essential for maintaining DC function regardless of the tissue O_2_ tension. In addition, our data suggest that the p62/RIP1 interaction not only contributes to pro-inflammatory signals but also participates in the establishment of DC hypoxic signature, as evidenced by the XRK3F2-mediated reduction in HIF-1α accumulation, which is known to be sustained by TLR4-dependent signals [19].

## Conclusion

In conclusion, our study identifies a novel mechanism by which p62, through its interaction with RIP1, regulates TLR4-mediated inflammatory responses in DCs. Such a role was active and relevant even in a hypoxic context, suggesting that it may remain unaffected by O_2_ availability, highlighting its broad significance in DC biology and suggesting potential therapeutic targets for modulating DC function in various inflammatory settings.

## Supplementary Information

Below is the link to the electronic supplementary material.

Supplementary file1 (DOCX 152 KB)

## Data Availability

The data generated during the current study and the material are available from the corresponding author on reasonable request.
