# Supplementary material for: Sequestosome-1/p62 Mediates TLR4-Induced Inflammatory Program in Dendritic Cells Under Normoxic and Hypoxic Conditions
Source: Cell Mol Life Sci. 2025 Dec 1;83(1):27. doi: 10.1007/s00018-025-05989-y (PMC12775214; doi:10.1007/s00018-025-05989-y)
Supplement: Supplementary file 1 — Supplementary file1 (DOCX 152 KB) [file 18_2025_5989_MOESM1_ESM.docx]

**
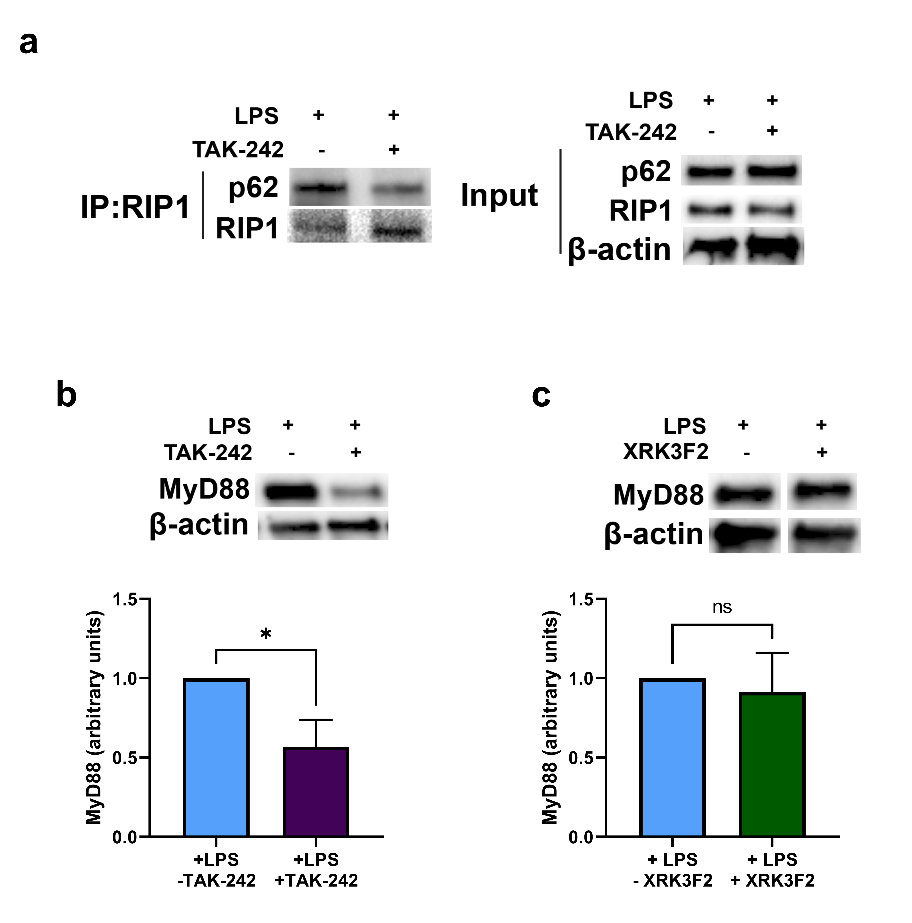
Supplementary Fig. 1**

**Fig. S1 (a)** Representative blot of co-immunoprecipitation of RIP1 with p62 and the relative input and **(b)** MyD88 protein levels, as shown by Western Blotting, in DCs pre-treated or not with TAK-242 (200 nM), stimulated with LPS and exposed to normoxia for 24h. **(c)** MyD88 protein levels, as determined Western blotting in DCs stimulated with LPS, exposed to normoxia for 24 h and treated or untreated during the last 6 h of incubation with XRK3F2. All blots shown are representative of at least three independent experiments and β-actin was used as loading control. β-actin was used as a housekeeping gene for RT-qPCR analysis. * indicates statistically significant differences (*p <* 0.05)
